# *Cryptococcus gattii* meningitis in an immunocompromised patient in a hospital in the Peruvian Amazon: case report

**DOI:** 10.17843/rpmesp.2025.422.14195

**Published:** 2025-06-12

**Authors:** Angel A. Moreno-Soto, Rodrigo J. Cardenas-Golac, Marco F. Paredes-Obando, Jhosephi J. Vasquez-Ascate, Jorge Sibina-Vela, Edgar A. Ramírez-García, Juan C. Celis-Salinas, Wilfredo M. Casapia-Morales

**Affiliations:** ¹ National University of the Peruvian Amazon, Iquitos, Peru. National University of the Peruvian Amazon National University of the Peruvian Amazon Iquitos Peru; ² Loreto Regional Hospital, Iquitos, Peru. Loreto Regional Hospital Iquitos Peru

**Keywords:** Cryptococcosis, immunocompromised host, amphotericin B.

## Abstract

We report a case of *Cryptococcus gattii* meningitis in a patient with HIV in the Peruvian Amazon. A 36-year-old male patient with severe neurological symptoms that was diagnosed by cerebrospinal fluid culture. Although liposomal amphotericin B and flucytosine are considered the standard antifungal therapy, due to a lack of resources, an alternative treatment of amphotericin B deoxycholate and fluconazole was used. Even with this alternative, treatment faced challenges due to the persistence of the microorganism. This case highlights the importance of considering *C. gattii* in the differential diagnosis of cryptococcal meningitis in immunocompromised patients, even in areas where the prevalence of this pathogen is low. The effectiveness of treatment and the patient’s survival underscore the need for diagnostic and therapeutic strategies adapted to resource-limited settings.

## INTRODUCTION

*Cryptococcus* species are encapsulated yeasts found in soil and bird feces. These microorganisms mainly affect individuals with compromised immune systems ^1^. There are 37 known species of *Cryptococcus*, but only *C. neoformans* and *C. gattii* are pathogenic. Infections caused by *C. gattii* are quite rare ^2^. The mortality rate in patients affected by this pathogen is approximately 14% ^3^.

Cryptococcal meningitis is a severe disease worldwide that mainly affects HIV-positive patients with low CD4 counts and those on immunosuppressive therapy. It is often caused by *C. neoformans* and, to a lesser extent, *C. gattii*^4^^,^^5^. Reports show that one million cases are diagnosed each year, resulting in more than 600,000 deaths ^6^.

Infection with *C. gattii* can affect immunocompetent and immunocompromised hosts. These may be asymptomatic until immunosuppressive factors, such as corticosteroid treatment or HIV infection, facilitate the manifestation of symptoms ^7^. The ability of C*. gattii* to cause disease in individuals with intact immune systems is due to its rapid replication in phagocytes before the adaptive immune response is activated ^8^. We report the first case of meningitis caused by *C. gattii* in an immunocompromised patient in the Peruvian Amazon.

## CASE REPORT

**Figure 1:**
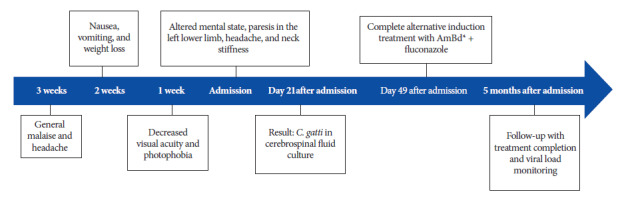
Timeline. *AmBd: Amphotericin B deoxycholate.

A 36-year-old male patient, born and raised in Iquitos, Peru, with high-risk sexual behavior and no other relevant medical history, was admitted to the emergency department of the Loreto Regional Hospital with three weeks of general malaise and moderate, throbbing frontal headache. Two weeks prior to admission, he experienced nausea, vomiting, and weight loss. One week prior, he reported decreased visual acuity and subjective photophobia (Figure 1).

During the physical examination, the patient was hemodynamically stable, weighing 71 kg, and had paresis in the left lower limb and severe headache. Neurological assessment showed altered mental status and neck stiffness, although cranial nerves were within normal limits. No abnormalities were detected on additional systemic examinations.

**Figure 2:**
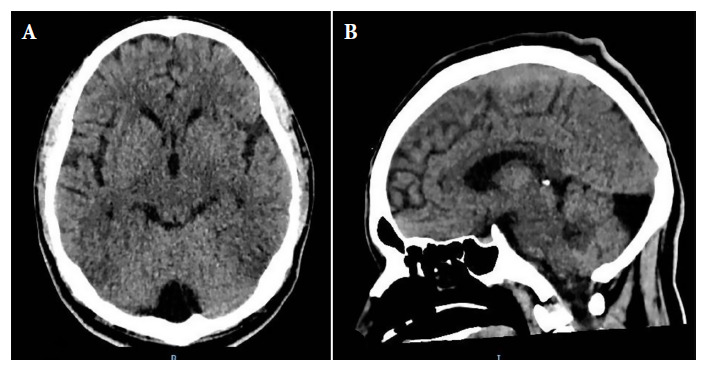
Non-contrast brain CT scan. Axial (A) and sagittal (B) views show a rounded, hypodense image with liquid density located in the midline of the posterior fossa, suggesting an arachnoid cyst. The rest of the brain parenchyma appears normal.

HIV infection was confirmed on admission by using a rapid test, subsequently validated by a PCR test, which showed a viral load of 207,000 cells/mL and a CD4 level of 34 cells/mL. The complete blood count showed lymphocytopenia with 800 cells/µL and mild anemia with hemoglobin of 11.4 g/dL. A cerebrospinal fluid (CSF) sample was obtained by lumbar puncture revealing an opening pressure of 30 cm/H2O, leukocytes of 6 cells/mL, glucose of 16.2 mg/dL, proteins of 40.9 mg/dL, and *Cryptococcus* spp. was found by India ink staining (Table 1). A brain CT scan showed an incidental finding of an arachnoid cyst in the posterior fossa (Figure 2).

**Table 1:** Laboratory tests on cerebrospinal fluid sample.

CSF	2 days	14 days	19 days	25 days
Leucocytes	6 cel/mm^3^	2 cel/mm^3^	2 cel/mm^3^	3 cel/mm^3^
Protein	40.9 mg/dL	43 mg/dL	56.3 mg/dL	59.7 mg/dL
Glucose	16.2 mg/dL	35.5 mg/dL	34 mg/dL	37.1 mg/dL
India ink	Positive	Positive	Positive	Positive
Bk staining	Negative	Negative	Negative	Negative
Gram staining	Negative	Negative	Negative	Negative
Opening Pressure	30 cm/H_2_O	75 cm/H_2_O	58 cm/H_2_O	38 cm/H_2_O
Culture result	-	Positive (1°LP)	Negative	Negative

*LP: lumbar puncture

Given the positive HIV results and the neurological abnormalities found during physical examination, additional tests were conducted. These showed a complete blood count with lymphocytopenia and mild anemia. In addition, a Sabouraud agar culture of a CSF sample isolated *Cryptococcus gattii* within 36 hours, with automated identification using VITEK® 2 Compact.

The patient was hospitalized and treated for cryptococcal meningitis. Antifungal therapy started with amphotericin B deoxycholate at a dose of 50 mg every 24 hours intravenously, plus fluconazole 800 mg daily orally. Adequate fluid and electrolyte replacement was provided before and after each dose. During the first two weeks of induction therapy, therapeutic lumbar punctures were conducted intermittently. The opening pressure was persistently high, with altered cellularity and biochemistry, as well as a sluggish clinical course. CSF culture results showed *C. gattii*. Treatment was extended for an additional two weeks, and the patient experienced significant clinical improvement. Consequently, upon discharge, consolidation therapy started with fluconazole 400 mg orally every 24 hours for 10 weeks.

One week after discharge, the patient attended a follow-up appointment at the infectious disease’s outpatient clinic, showing clinical improvement. He was instructed to continue treatment with fluconazole 400 mg and cotrimoxazole 800/160 mg orally every 24 hours for 10 weeks. Five months later, at a follow-up visit, a viral load of <40 copies of RNA was confirmed.

## DISCUSSION

Upon admission to the hospital, the patient was diagnosed with cryptococcal meningitis and HIV infection, leading to the immediate start of antifungal treatment. During the disease course, the sluggish clinical response suggested that the causative agent was probably not *Cryptococcus neoformans*. After two weeks of induction therapy, and during hospitalization, a culture result showed *Cryptococcus gattii*.

The low prevalence of *C. gattii* compared to *C. neoformans*, particularly in immunocompromised patients such as those with HIV infection, makes diagnosis difficult ^9^. However, recent outbreaks in North America and Australia have expanded the known risk groups to include patients with cancer, solid organ transplants, and other immunodeficiencies ^10^^,^^11^. In Peru, there is evidence of cases of cryptococcosis caused by *C. neoformans* var. *gatti* (which remains a variant of *C. neoformans* itself) ^12^, but only one case has been reported for *C. gatti* as a variety, and that was in the Peruvian highlands in an immunocompetent patient ^13^, unlike this report, in which the patient was immunocompromised.

It is recommended to perform a CSF culture after 2 weeks of induction therapy to assess sterility if clinical symptoms persist, which serves as an indicator of therapy success before proceeding to the consolidation phase ^14^. This is consistent with our report, in which the difference between the initial positive culture and the subsequent culture, which was negative, was 12 days, similar to the case of Gutierrez *et al*., where culture sterility was achieved on day 19 ^13^.

Current treatment guidelines recommend the use of liposomal amphotericin B and flucytosine to treat cryptococcal meningitis caused by *C. gattii*, with a treatment regimen of 4 to 6 weeks ^15^^,^^16^. However, in resource-limited settings where these drugs are not available, induction therapy with amphotericin B deoxycholate (AmBd) at a dose of 1 mg/kg per day IV or a combination of AmBd at 0.7 mg/kg per day IV plus fluconazole at 800 mg per day orally is suggested ^9^. This approach was used by Lizarazo *et al*. in Colombia, who, in the absence of flucytosine, used amphotericin B deoxycholate together with fluconazole in 90 patients ^17^, similarly to the study by Gutierrez *et al*^13^.

The use of liposomal amphotericin B over deoxycholate is preferred due to the lower probability of developing nephrotoxicity, although studies have shown that its efficacy at 2 and 10 weeks is comparable to the liposomal presentation ^18^. Nevertheless, in our report, at 28 days, the patient received a cumulative dose of 1400 mg of AmBd, unlike the 535 mg reached in the report by Gutierrez *et al*, in which the patient only received 11 days of the drug ^13^. Despite the cumulative dose used in our patient, he did not develop nephrotoxicity. In contrast, a patient in Cuba with the same diagnosis and etiological agent, also treated with AmBd and fluconazole, presented nephrotoxicity after a cumulative dose of 1500 mg of AmBd, which forced a change in treatment to the liposomal formulation of amphotericin B ^16^.

The diagnosis of cryptococcosis caused by *C. gattii* is made through a combination of clinical evaluation, physical examination, and additional tests. The cryptococcal antigen test is the most sensitive, but it does not distinguish between *C. gattii* and *C. neoformans*, so culture remains essential for a definitive diagnosis that differentiates between these species ^19^. Initial neurological symptoms often include headache and neck stiffness. As the disease progresses, other neurological signs may appear, such as seizures, cranial nerve abnormalities, cerebellar irregularities, focal weakness in the limbs, and changes in mental status. The average time from the onset of the first symptoms to diagnosis is approximately 45 days ^3^. In this case, the initial diagnosis was made at 21 days, indicating probable *C. neoformans* meningitis, but was later adjusted to *C. gattii* after isolation of the pathogen. The patient presented general symptoms, like malaise, progressive headache, nausea, and vomiting. One week before admission, he also experienced decreased visual acuity and photophobia.

The case illustrates unique diagnostic and therapeutic challenges associated with *C. gattii*, highlighting the need for a more intensive and prolonged treatment approach in the presence of severe neurological complications. It also emphasizes the importance of accurate diagnosis through culture to guide treatment and improve clinical outcomes. Furthermore, it highlights the importance of medical awareness and continuing education on the diversity of cryptococcosis in different populations, reaffirming the need for epidemiological surveillance and adaptability in clinical management strategies to optimize care and health outcomes in patients with invasive fungal infections.

In conclusion, *Cryptococcus gattii* should be considered in the differential diagnosis of meningitis in HIV-positive patients with neurological symptoms who do not respond to initial induction therapy, even in regions where its prevalence is low. Increasing awareness of this rare infection and its therapeutic challenges is crucial to improving clinical outcomes in these vulnerable populations.

## References

[1] Kenosi K, Mosimanegape J, Daniel L, Ishmael K (2023). Recent advances in the ecoepidemiology, virulence and diagnosis of Cryptococcus neoformans and Cryptococcus gattii species complexes. Open Microbiol J.

[2] Perfect JR, Casadevall A, Heitman J, Kozel TR, Kwon-Chung J, Perfect J (2011). The history of Cryptococcus and Cryptococcosis, Cryptococcus: From Human Pathogen to Model Yeast, 2011.

[3] Chen SC, Slavin MA, Heath CH, Playford EG, Byth K, Marriott D (2012). Manifestaciones clínicas de la infección por Cryptococcus gattii determinantes de secuelas neurológicas y muerte. Clin Infect Dis.

[4] Bruner KT, Franco-Paredes C, Andrés F, Henao-Martínez AF, Steele GM, Chastain DB (2018). Cryptococcus gattii Complex Infections in HIV-Infected Patients, Southeastern United States. Emerg Infect Dis.

[5] Hurtado-Bedoya JD, Riveros Santoya SV (2023). Criptococosis meníngea características y desenlace clínico en un hospital de tercer nivel de Bogotá, Colombia. Acta Neurol Colomb.

[6] Park BJ, Wannemuehler KA, Marston BJ, Govender N, Pappas PG, Chiller TM (2009). Estimation of the current global burden of cryptococcal meningitis among persons living with HIV/AIDS. AIDS.

[7] Springer DJ, Billmyre RB, Filler EE, Voelz K, Pursall R, Mieczkowski PA, Larsen RA (2014). Cryptococcus gattii VGIII isolates causing infections in HIV/AIDS patients in Southern California identification of the local environmental source as arboreal. PLoS Pathog.

[8] Okudo J, Civelli VF, Narang VK, Johnson RH, Khan N, Andruszko B (2020). A Rare Case of Cryptococcus gattii Meningitis in Advanced HIV Disease, Sagittal Thrombosis, and Immune Reconstitution Syndrome, Resolved With Isavuconazonium. J Investig Med High Impact Case Rep. enero de.

[9] Franco-Paredes C, Womack T, Bohlmeyer T, Sellers B, Hays A, Patel K (2015). Manejo de la meningoencefalitis por Cryptococcus gattii. Lancet Infect Dis.

[10] Perfect JR, Dismukes WE, Dromer F, Goldman DL, Graybill JR, Hamill RJ (2010). Clinical practice guidelines for the management of cryptococcal disease 2010 update by the Infectious Diseases Society of America. Clin Infect Dis.

[11] Byrnes EJ III, Bartlett KH, Perfect JR, Heitman J (2011). Cryptococcus gattii an emerging fungal pathogen infecting humans and animals. Microbes Infect.

[12] Rufino B, Swinne D (1998). Aislamiento de Cryptococcus neoformans variedad gattii en dos pacientes peruanos. Rev Iberoam Micol.

[13] Gutierrez EL, Valqui W, Vilchez L, Evangelista L, Crispin S, Tello M (2010). Cryptococcus gattii meningoencephalitis in an HIV-negative patient from the Peruvian Andes. Rev Soc Bras Med Trop.

[14] Day JN, Chau TTH, Wolbers M, Mai PP, Dung NT, Mai NH (2013). Combination antifungal therapy for cryptococcal meningitis. N Engl J Med.

[15] Chen SC-A, Meyer W, Sorrell TC (2014). Cryptococcus gattii infections. Clin Microbiol Rev.

[16] Illnait-Zaragozí MT, Ortega-Gonzalez LM, Hagen F, Martínez-Machin GF, Meis JF (2013). Fatal Cryptococcus gattii genotype AFLP5 infection in an immunocompetent Cuban patient. Med Mycol Case Rep.

[17] Lizarazo J, Chaves O, Peña Y, Escandón P, Agudelo CI, Castañeda E (2012). Comparación de los hallazgos clínicos y de supervivencia entre pacientes VIH positivos y VIH negativos con criptococosis meníngea en un hospital de tercer nivel. Acta Med Colomb.

[18] Hamill RJ, Sobel JD, El-Sadr W, Johnson PC, Graybill JR, Javaly K (2010). Comparison of 2 doses of liposomal amphotericin B and conventional amphotericin B deoxycholate for treatment of AIDS-associated acute cryptococcal meningitis A randomized, double-blind clinical trial of efficacy and safety. Clin Infect Dis.

[19] Chang CC, Harrison TS, Bicanic TA, Chayakulkeeree M, Sorrell TC, Warris A (2024). Global guideline for the diagnosis and management of cryptococcosis an initiative of the ECMM and ISHAM in cooperation with the ASM. Lancet Infect Dis.

