# Supplementary material for: Cryptococcus gattii meningitis in an immunocompromised patient in a hospital in the Peruvian Amazon: case report
Source: Rev Peru Med Exp Salud Publica. 2025 Jun 12;42(2):212–6. doi: 10.17843/rpmesp.2025.422.14195 (PMC12377883; doi:10.17843/rpmesp.2025.422.14195)
Supplement: Translation

# Translation: Meningitis por *Cryptococcus gattii* en paciente inmunodeprimido en un Hospital de la Amazonía peruana: reporte de caso

**Authors:** Angel A. Moreno-Soto, Rodrigo J. Cardenas-Golac, Marco F. Paredes-Obando, Jhosephi J. Vasquez-Ascate, Jorge Sibina-Vela, Edgar A. Ramírez-García, Juan C. Celis-Salinas, Wilfredo M. Casapia-Morales

**Affiliations:** 1 National University of the Peruvian Amazon, Iquitos, Peru.; 2 Loreto Regional Hospital, Iquitos, Peru.

**Keywords:** Cryptococcosis, huesped inmunocomprometido, anfotericina

## Abstract

Reportamos un caso de meningitis por *Cryptococcus gattii* en un paciente con VIH en la Amazonía peruana. Paciente varón de 36 años, presentó síntomas neurológicos severos y fue diagnosticado por cultivo de líquido cefalorraquídeo. A pesar de que anfotericina B liposomal y flucitosina son consideradas la terapia antifúngica estándar, ante la falta de recursos se utilizó un régimen alterno de anfotericina B desoxicolato y fluconazol, e incluso con ello, el tratamiento enfrentó desafíos debido a la persistencia del microorganismo. Este caso resalta la importancia de considerar a *C. gattii* en el diagnóstico diferencial de meningitis criptocócica en pacientes inmunodeprimidos, incluso en áreas donde la prevalencia de este patógeno es baja. La efectividad del tratamiento y la supervivencia del paciente destacan la necesidad de estrategias diagnósticas y terapéuticas adaptadas a entornos con recursos limitados.

## INTRODUCCIÓN

Las especies de *Cryptococcus* son levaduras encapsuladas que se encuentran tanto en el suelo como en las heces de las aves. Estos microorganismos afectan principalmente a individuos con sistemas inmunológicos comprometidos ^1^. Se conocen 37 especies de *Cryptococcus*, pero solo *C. neoformans* y *C. gattii* son patógenos. Las infecciones por *C. gattii* son bastante raras ^2^. La tasa de mortalidad en pacientes afectados por este patógeno es del 14% aproximadamente ^3^.

A nivel mundial, la meningitis criptocócica es una enfermedad grave que afecta principalmente a pacientes VIH positivos con bajos recuentos de CD4 y a aquellos en terapia inmunosupresora, con frecuencia suele ser causada por *C. neoformans*, y en menor medida *C. gattii*^4^^,^^5^. Se estima que cada año se diagnostican un millón de casos, resultando en más de 600,000 muertes ^6^.

La infección por *C. gattii* puede afectar a huéspedes inmunocompetentes e inmunocomprometidos. Estos pueden ser asintomáticos hasta qué factores inmunosupresores, como la administración de corticosteroides o una infección por VIH, faciliten la manifestación de síntomas ^7^. La capacidad de *C. gattii* para enfermar a individuos con sistemas inmunes intactos se debe a su rápida replicación en los fagocitos antes de la activación de la respuesta inmune adaptativa ^8^. Reportamos el primer caso de meningitis causada por *C. gattii* en un paciente inmunodeprimido, en la Amazonia peruana.

## REPORTE DE CASO

Paciente varón de 36 años, natural y procedente de Iquitos, Perú, con conducta sexual de alto riesgo, sin otros antecedentes médicos relevantes. Acudió al servicio de emergencias del Hospital Regional de Loreto, presentando tres semanas de malestar general y cefalea frontal opresivo punzante de moderada intensidad. Dos semanas antes del ingreso, se acompañada de náuseas, vómitos y pérdida de peso, una semana antes, reportó disminución de la agudeza visual y fotofobia subjetiva (Figura 1).

**Figura 1:** Línea de tiempo. *AmBd: Anfotericina B deoxicolato

Durante el examen físico, paciente hemodinámicamente estable, con un peso de 71 kg, mostró paresia en el miembro inferior izquierdo y cefalea intensa. La evaluación neurológica indicó un estado mental alterado y rigidez de nuca, aunque los pares craneales se encontraban dentro de los parámetros normales. No se detectaron anomalías en los exámenes sistémicos adicionales.

Al ingreso, se confirmó la infección por VIH usando una prueba rápida, validada posteriormente por una prueba de PCR, que mostró una carga viral de 207,000 células/mL y un nivel de CD4 de 34 células/mL. En el hemograma, se observó linfocitopenia con 800 células/µL y anemia leve con hemoglobina de 11,4 g/dL. Se realizó una punción lumbar, obteniéndose muestra de líquido cefalorraquídeo (LCR) con presión de apertura de 30 cm/H2O, leucocitos de 6 células/mL, glucosa de 16,2 mg/dL, proteínas de 40,9 mg/dL, y se observó *Cryptococcus spp.* por tinción de tinta china (Tabla 1). La tomografía computarizada cerebral mostró hallazgo incidental de quiste aracnoideo en fosa posterior (Figura 2).

**Tabla 1:** Exámenes laboratoriales en muestra de líquido cefalorraquídeo.

LCR	2 días	14 días	19 días	25 días
Leucocitos	6 cel/mm^3^	2 cel/mm^3^	2 cel/mm^3^	3 cel/mm^3^
Proteína	40,9 mg/dL	43 mg/dL	56,3 mg/dL	59,7 mg/dL
Glucosa	16,2 mg/dL	35,5 mg/dL	34 mg/dL	37,1 mg/dL
Tinta China	Positivo	Positivo	Positivo	Positivo
Tinción Bk	Negativo	Negativo	Negativo	Negativo
Tinción Gram	Negativo	Negativo	Negativo	Negativo
Presión Apertura	30 cm/H_2_O	75 cm/H_2_O	58 cm/H_2_O	38 cm/H_2_O
Resultado Cultivo	-	Positivo (1°PL)	Negativo	Negativo

*PL: punción lumbar

**Figura 2:** Tomografía cerebral sin contraste. A Cortes axial y B) sagital muestran una imagen redondeada e hipodensa, de densidad líquida, localizada en la línea media de la fosa posterior, sugiriendo un quiste aracnoideo. El resto del parénquima cerebral presenta características normales.

Ante los resultados positivos para VIH y las alteraciones neurológicas observadas en el examen físico, se realizaron exámenes complementarios. Estos mostraron un hemograma con linfocitopenia y anemia leve. Además, un cultivo en agar Sabouraud de una muestra de LCR aisló *Cryptococcus gattii* en 36 horas, con identificación automatizada mediante VITEK^®^ 2 Compact.

El paciente fue hospitalizado y tratado por meningitis criptocócica. Se inició terapia antifúngica con anfotericina B desoxicolato a una dosis de 50 mg cada 24 horas por vía intravenosa, más fluconazol 800 mg al día vía oral. Se aseguró una adecuada reposición hidroelectrolítica antes y después de cada administración. Durante las primeras dos semanas de terapia de inducción, se realizaron punciones lumbares terapéuticas de manera intermitente. Se observó la persistencia de una presión de apertura elevada, con celularidad y bioquímica alteradas, además de una evolución clínica tórpida. Se recibió los resultados del cultivo de LCR, aislándose *C. gattii*. Se prolongó el tratamiento por dos semanas adicionales, y el paciente experimentó una mejoría clínica significativa. Consecuentemente, al alta médica, se inició con la terapia de consolidación utilizando fluconazol 400 mg oral cada 24 horas durante 10 semanas.

A la semana del alta, el paciente acudió a la consulta de control en el consultorio externo de infectología, evidenciando una mejoría clínica. Se le indicó continuar el tratamiento con fluconazol 400 mg y cotrimoxazol 800/160 mg por vía oral cada 24 horas, hasta cumplir las 10 semanas. Cinco meses después, en una consulta de seguimiento, se constató una carga viral de <40 copias de ARN.

## DISCUSIÓN

Al ingresar al hospital, el paciente fue diagnosticado con meningitis criptocócica e infección por VIH, lo que llevó al inicio inmediato del tratamiento antifúngico. Durante su evolución, se observó una respuesta clínica tórpida, orientando a que el agente causante probablemente no era *Cryptococcus neoformans*. Después de dos semanas de terapia de inducción, y durante su hospitalización, se recibe resultado de un cultivo en el que se aisló *Cryptococcus gattii*.

La baja prevalencia de *C. gattii* en comparación con *C. neoformans*, particularmente en pacientes inmunocomprometidos como infección por VIH, dificulta la sospecha diagnóstica ^9^. Sin embargo, los brotes recientes en América del Norte y Australia han ampliado los grupos de riesgo conocidos para incluir a pacientes con cáncer, trasplantes de órganos sólidos y otras inmunodeficiencias ^10^^,^^11^. En el Perú, existen evidencia de casos de Cryptoccocsis por *C. neoformans* var. gatti (que sigue siendo una variante del mismo *C. neoformans*) ^12^, pero solo se ha reportado un caso por *C. gatti* como variedad y fue en la sierra peruana en un paciente inmunocompetente ^13^ a diferencia de este reporte donde el paciente estaba inmunocomprometido.

Ante la persistencia de síntomas clínicos, se recomienda realizar un cultivo del LCR después de 2 semanas de terapia de inducción para evaluar su esterilidad, lo cual sirve como un indicador del éxito de la terapia antes de proceder a la fase de consolidación ^14^. Esto concuerda con nuestro reporte, donde la diferencia entre el cultivo positivo inicial y el siguiente cultivo, que resultó negativo, fue de 12 días; similar al caso de Gutierrez *et al.* donde se logró la esterilidad del cultivo al día 19 ^13^.

Las pautas de tratamiento actuales recomiendan el uso de anfotericina B liposomal y flucitosina para tratar la meningitis criptocócica causada por *C. gattii*, con un régimen de tratamiento de 4 a 6 semanas ^15^^,^^16^. No obstante, en entornos con recursos limitados donde no hay disponibilidad de estos fármacos, se sugiere la terapia de inducción con anfotericina B desoxicolato (AmBd) a una dosis de 1 mg/kg por día IV o una combinación de AmBd a 0,7 mg/kg por día IV más fluconazol a 800 mg por día por vía oral ^9^. Este enfoque fue empleado por Lizarazo *et al*. en Colombia, quienes, ante la falta de flucitosina, utilizaron anfotericina B desoxicolato junto con fluconazol en 90 pacientes ^17^^)^ y de igual forma por Gutierrez *et al*^13^.

La utilización de anfotericina b liposoma sobre desoxicolato, es preferida debido a la menor probabilidad de desarrollar nefrotoxicidad, aunque los estudios han demostrado que su eficacia a las 2 y 10 semanas es comparada a la presentación liposomal ^18^. A pesar de ello, en nuestro reporte, a los 28 días, recibió una dosis acumulada de 1400 mg de AmBd, diferente a los 535 mg alcanzados en el reporte de Gutierrez *et al* que solo recibió 11 días del medicamento ^13^. A pesar de la dosis acumulativa usada en nuestro paciente, no llegó a presentar nefrotoxicidad. En contraste, un paciente en Cuba con el mismo diagnóstico y agente etiológico, tratado también con AmBd y fluconazol, presentó nefrotoxicidad a partir de una dosis acumulada de 1500 mg de AmBd, lo que obligó a cambiar el tratamiento a la presentación liposomal de la anfotericina B ^16^.

El diagnóstico de criptococosis causada por *C. gattii* se realiza mediante una combinación de evaluación clínica, examen físico y pruebas complementarias. La prueba de antígeno criptocócica es la más sensible, pero no distingue entre *C. gattii* y *C. neoformans*, por lo que el cultivo sigue siendo esencial para un diagnóstico definitivo que diferencie estas especies ^19^. Los síntomas neurológicos iniciales frecuentemente incluyen dolor de cabeza y rigidez de nuca. Con el avance de la enfermedad, pueden aparecer otros signos neurológicos como convulsiones, anomalías en los nervios craneales, irregularidades cerebelosas, debilidad focal en las extremidades y cambios en el estado mental. El tiempo promedio desde la aparición de los primeros síntomas hasta el diagnóstico es de aproximadamente 45 días ^3^. En este caso, el diagnóstico inicial se realizó a los 21 días indicando meningitis por *C. neoformans* probable, pero posteriormente se ajustó a *C. gattii* tras el aislamiento del patógeno. El paciente presentó síntomas generales de malestar, cefalea progresiva, náuseas y vómitos. Una semana antes de su admisión, también experimentó disminución de la agudeza visual y fotofobia.

El caso ilustra desafíos diagnósticos y terapéuticos únicos asociados con *C. gattii*, resaltando la necesidad de un enfoque de tratamiento más intensivo y prolongado en presencia de complicaciones neurológicas severas. También enfatiza la importancia de un diagnóstico preciso mediante cultivo para dirigir adecuadamente el tratamiento y mejorar los resultados clínicos. Además, pone de relieve la importancia de la conciencia médica y la educación continua sobre la diversidad de la criptococosis en diferentes poblaciones, reafirmando la necesidad de vigilancia epidemiológica y adaptabilidad en las estrategias de manejo clínico para optimizar la atención y los resultados de salud en pacientes con infecciones fúngicas invasivas.

En conclusión, *Cryptococcus gattii* debe considerarse en el diagnóstico diferencial de meningitis en pacientes VIH positivos con síntomas neurológicos, que no responden a la terapia de inducción inicial, incluso en regiones donde su prevalencia es baja. Aumentar el conocimiento sobre esta rara infección y sus desafíos terapéuticos es crucial para mejorar los resultados clínicos en estas poblaciones vulnerables.
